# Influence of a Virtual Plant-Based Culinary Medicine Intervention on Mood, Stress, and Quality of Life Among Patients at Risk for Cardiovascular Disease †

**DOI:** 10.3390/nu17081357

**Published:** 2025-04-16

**Authors:** Andrea M. Krenek, Monica Aggarwal, Stephanie T. Chung, Amber B. Courville, Nicole Farmer, Juen Guo, Anne Mathews

**Affiliations:** 1 Food Science and Human Nutrition Department, University of Florida, Gainesville, FL 32611, USA; akrenek@stanford.edu; 2 National Institute of Diabetes and Digestive and Kidney Diseases, National Institutes of Health, Bethesda, MD 20892, USA; stephanie.chung@nih.gov (S.T.C.); amber.courville@nih.gov (A.B.C.); juen.guo@nih.gov (J.G.); 3 Division of Cardiovascular Medicine, University of Florida, Gainesville, FL 32611, USA; monica.aggarwal@medicine.ufl.edu; 4 National Institutes of Health Clinical Center, Bethesda, MD 20892, USA; nicole.farmer@nih.gov

**Keywords:** culinary medicine, teaching kitchens, cardiovascular disease, psychological health, mood, stress

## Abstract

**Background**: Cooking and dietary intake may affect psychological well-being. **Objective**: We evaluated the effects of a virtual culinary medicine teaching kitchen intervention on psychosocial health. **Methods**: In a randomized crossover trial implementing a vegan diet high or low in extra virgin olive oil, adults with ≥5% atherosclerotic cardiovascular disease risk participated in eight weekly group cooking classes. Psychosocial survey assessments of perceived stress, positive and negative affect, and quality of life before and after the intervention were compared using paired *t*-tests and post hoc linear mixed models. **Results**: Pre-post analysis among 40 participants (75% female, 64.4 ± 8.6 years) indicated a 19% decrease in perceived stress (*p* < 0.01), 6–8% increase in positive affect (*p* < 0.04), and 13% decrease in negative affect (*p* = 0.02). Energy/fatigue and general health-related quality of life improved post-intervention (both *p* ≤ 0.02). **Conclusions**: Participation in a group culinary medicine intervention improved mood, stress, and health-related quality of life, warranting larger, diverse studies. Benefits may relate to social support, improved health status, diet factors, and emerging psychosocial influences of cooking.

## 1. Introduction

Excessive psychological stress and related mental health conditions, including depression and anxiety, are strongly implicated in pathological consequences. Psychological stressors can lead to cardiometabolic disorders, among other chronic diseases [1,2], and the American Heart Association emphasizes the importance of negative psychological factors on leading causes of death, such as cardiovascular disease [3]. Stressors may further trigger risk-enhancing behaviors, such as poor nutrition habits and reduced physical activity, though this relationship may be bidirectional. While lower stress levels are connected to healthier eating habits, increased stress may lead to or be a result of an increased intake of less optimal foods [4,5]. A range of research efforts are devoted towards developing both pharmaceutical and lifestyle behavioral interventions for reducing the burden of disease connected to mental and cognitive disorders. Nutrition, cooking, physical activity, social support, sleep, and stress management are among lifestyle factors considered in treatment interventions that may influence cognitive and psychological well-being [6,7,8,9,10].

Increasing evidence on the role of nutrition in mental health and the field of nutritional psychiatry has emphasized potential benefits of plant-based dietary patterns rich in fruits and vegetables [11,12,13]. These patterns include Mediterranean diets [14,15], healthful vegetarian and vegan diets [16,17], and low-inflammatory dietary patterns [18,19], which may lower disease risk and mortality [20,21]. However, conflicting data exist on the investigation of associations between vegan diets and mental health [22] in relation to positive, negative, or neutral correlations. Plant-based patterns that emphasize fruits, vegetables, other sources of dietary fiber, and unsaturated fatty acids may support mood improvements perhaps in part through inflammatory pathways and gut–brain communication. The consumption of at least 5–9 servings of fruits and vegetables may promote higher levels of positive affect, optimism, self-efficacy, and well-being while protecting against psychological distress and depressive symptoms [12]. In a randomized controlled trial of adults with low habitual vegetable consumption, Leon et al. showed greater measures of happiness after 8 weeks of increased vegetable consumption [23]. Moreover, higher diet quality, as assessed by greater adherence to an overall and healthful plant-based diet index, has been associated with lower odds of depression and anxiety (OR: 0.51, 95% CI: 0.38–0.68) when comparing the highest to lowest quintiles of consumption [13]. This is reflective of another cross-sectional analysis indicating a greater likelihood of mental health disorders and poor sleep with animal-based or unhealthful plant-based dietary patterns [24]. When omnivorous participants in another clinical trial were randomized to a control group consuming meat, fish, and poultry, a comparator group consuming fish 3–4 times weekly without meat and poultry, and a third vegetarian intervention arm avoiding all three animal foods, improvements in mood scores were only detected in the vegetarian group [25]. As emphasized to a greater extent in Mediterranean patterns than standard vegetarian diets, extra virgin olive oil has further been proposed as a possible factor that influences depression [26] and is potentially related to interactions of predominant fatty acids (i.e., oleic acid) on serotonin and cortisol levels [3,11,12,13,14,15,16,17,18,19,20,21,22,27,28,29]. As one avenue of influencing nutritional intake, cooking interventions have further been linked to psychosocial and cognitive health [30] as well as outlined as a framework for well-being [31], with potential benefits beyond affecting diet. By implementing the development of cooking skills, healthful nutrition habits, and social relationships, culinary medicine in teaching kitchen settings has emerged as a novel approach for influencing physical and mental health status as well as clinical care and research.

Culinary medicine (CM) describes the blending of the art of preparing food with the science of medicine to address underlying disease processes while maintaining the sensory enjoyment of food [32,33,34]. Teaching kitchens, which allow for the experiential practice of lifestyle-related skills while supporting the adherence of behavior change, are increasingly used for applying CM, often through group classes or shared medical appointments when in clinical environments [35,36,37]. Multiple practice applications within CM sessions may influence behavioral determinants that affect psychological well-being. The prominent well-being model PERMA (Positive emotion, Engagement, Relationships, Meaning, and Accomplishment) has further been proposed as a determinant for evaluating the connection between cooking and well-being [31]. Cooking as a means of expressing creativity and practicing mindfulness may additionally support positive mood changes [38]. As loneliness and social isolation can further exacerbate negative mental health and chronic disease risk as a strong predictor of premature mortality [3,28,29], group cooking interventions may offer a source of community.

While the impact of nutritional habits on psychological health remains equivocal, limited preliminary studies have evaluated the influence of CM on stress and mental well-being. This paper describes the psychological outcomes of the Recipe for Heart Health CM group teaching kitchen study, which implemented a whole food, plant-based vegan diet pattern intervention, as related to perceived stress, positive and negative affect, and quality of life. As previously reported, this study intervention resulted in improvements in cardiometabolic, cooking competency, and dietary factors; here, we assessed pre–post changes in mood-related measures from this teaching kitchen intervention. We hypothesized improvements in affect and quality of life, with unchanged perceived stress among participants after the intervention compared to baseline.

## 2. Materials and Methods

This study protocol was approved by the University of Florida Institutional Review Board (IRB202002194). All study participants provided written informed consent obtained by investigators or trained study coordinators prior to any study procedures.

### 2.1. Study Design and Sample

Recipe for Heart Health was a randomized, controlled culinary medicine crossover clinical trial (NCT04828447) of primary prevention adults randomly assigned to follow a vegan diet high (4 tablespoons/day) or low (<1 tsp/day) in extra virgin olive oil (EVOO) for four weeks each, separated by a 1-week washout period [39] Eligible participants included adult patients (≥18 years up to age 79) with borderline to high risk for atherosclerotic cardiovascular disease (ASCVD), as indicated by a 10-year risk score (>40 years) or lifestyle ASCVD risk (if younger than 40 years, n = 2) of ≥5% according to the American College of Cardiology/American Heart Association ASCVD Risk Calculator. Secondary prevention patients, pregnant women, and those with defined metabolic abnormalities or that are currently following a Mediterranean or vegan diet pattern were excluded. The primary outcome was low-density lipoprotein cholesterol (LDL-C) that informed the power calculation, with pre-defined secondary analyses of psychosocial measures.

### 2.2. Culinary Medicine Intervention

A registered dietitian/chef met individually with participants to describe the vegan diet pattern intervention and to provide individualized meal planning prior to leading eight virtual 90 min teaching kitchen sessions by Zoom™ (versions 5.6.0 to 5.10.0). The dietary pattern was characterized by the emphasis on foods within categories of whole fruits, vegetables/legumes, whole grains, and nuts/seeds. Participants were asked to avoid animal products (meat, poultry, fish and other seafood, dairy, and eggs) in addition to heavily processed or refined foods items with added sugars, oils, or refined grains. All extra virgin olive oil was provided to consume in its raw state. Balanced meals were encouraged without the specification of controlled caloric or macronutrient composition.

With hands-on cooking practice, culinary medicine sessions consisted of facilitated group discussion on culinary topics, nutrition education, and lifestyle behavior skills with group check-ins, demonstrations, and active recipe preparation and taste-testing. Teaching kitchen curricula content has been detailed previously [40]. Recipe, didactic, and experiential support were provided throughout the study in addition to a variety of resources, including a research study cookbook, articles, podcasts, videos, and additional group engagement through frequent pre-class and post-class email communication. Foods prepared aligned with the advised dietary intervention in the study cookbook, which also included pages to record weekly questions, challenges, successes, planning, and goals.

### 2.3. Outcomes

Psychological survey measures were assessed at time points before and after two 4-week diet interventions in addition to metabolic, clinical, and dietary outcomes previously reported. To evaluate areas of mood and well-being, participants completed survey assessments of positive and negative affect [41,42], perceived stress [43], and quality of life [44]. The RAND 36-Item Short Form Health Survey, with subscales for eight areas of health, including general health, physical health problems, energy/fatigue, social functioning, and emotional well-being, was used to evaluate patient quality of life well-being. Items scored on a 0 to 100 scale were averaged to create scale scores, with higher scores corresponding to a more favorable health state. Self-perceived changes in stress levels were evaluated using the widely administered 10-item Perceived Stress Scale (PSS), which applies a 5-point Likert scale [43]. PSS scores range from 0 to 40, with higher scores indicating higher perceived stress. Items on the Positive and Negative Affect Schedule (PANAS) were used to assess emotional status, with 10 items representing levels of positive affect and 10 items corresponding to negative affect on a 10–50-point scale. Participants responded to PANAS items and were instructed to indicate the extent to which they generally feel on the average, and they were asked after each diet period over the prior month. To further explore capturing emotions that reflect “pleasurable engagement with the environment, such as happiness, joy, excitement, enthusiasm, and contentment,” participants responded to questions from the expanded bank of positive affect questions from the NIH Toolbox Emotion Measures common data elements [45].

### 2.4. Statistical Analysis

Statistical analyses were performed using IBM SPSS Statistics 29.0 and SAS 9.4. Paired *t*-tests for parametric variables or Wilcoxon signed rank tests for non-normal variables were used to analyze changes in scores from baseline for perceived stress, positive and negative affect, and quality of life before and after completing the culinary medicine intervention. The study sample size was powered based on prior data evaluating the primary outcome, LDL-C [39]. Post hoc comparisons of psychological outcomes by diet randomization (high vs. low extra virgin olive oil) were analyzed by linear mixed models adjusted for age, sex, and body weight change. All statistical tests were two-sided, with significance considered at the *p* < 0.05 level.

## 3. Results

### 3.1. Demographics

Sample characteristics are described in Table 1. Participants were pooled across initial randomization groups for pre–post analyses, overall receiving the same EVOO exposure during the 9-week crossover intervention. Most participants had obtained at least a college degree (65%), were primarily responsible for cooking meals (80%), and had reported sleeping between 6 and 8 h daily (87.5%). Each group cooking class included 3–15 individuals in attendance within six cohorts over an 11-month time period. All participants completed all eight cooking sessions. A detailed analysis of dietary data [40], collected by dietary recalls, objective measures of animal food intake (via trimethylamine n-oxide) and fruit/vegetable intake (via skin carotenoid status), and weekly verbal confirmations at cooking classes affirmed dietary adherence.

### 3.2. Stress, Well-Being, and Quality of Life

**Figure 1:**
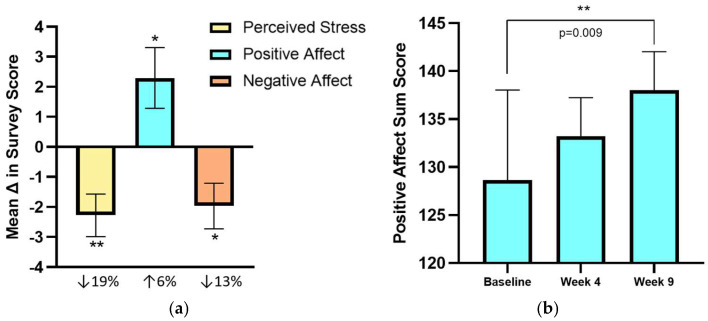
(**a**) Changes in mood and stress survey score measures for Recipe for Heart Health participants from baseline to post-intervention for perceived stress, positive affect, and negative affect. (**b**) Positive affect sum scores at baseline, week 4, and week 9. Values are presented as mean ± SEM. Paired *t*-tests analyzed changes in survey scores. * *p* ≤ 0.05, ** *p* ≤ 0.01.

Figure 1 shows changes in measures of perceived stress, positive and negative affect, and negative affect collected at baseline and post-intervention [46]. Participant baseline stress levels ranged from 2 to 32 (corresponding to low to high stress), with a mean of 13, the uppermost bound of low stress before indicating moderate stress. Pre–post analysis indicated that perceived stress significantly decreased (−2.3 ± 0.7, *p* = 0.002) after the intervention in contrast to baseline reports. Additionally, positive affect increased by ±6.4% while negative affect decreased by −13%, considering PANAS survey responses at the same time points (*p* = 0.044 and *p* = 0.019). Mean baseline scores of positive and negative affect among participants were 35.2 and 15.6, respectively. Separate NIH toolbox positive affect questions further significantly increased from 128.7 ± 4.4 to 138.0 ± 4.0 as sum values (Figure 1b).

Considering post hoc comparisons between diets, there was a greater change in negative affect from baseline to post-intervention among participants who started with the low EVOO before transitioning to high EVOO (*p* = 0.03). Positive affect increased more during the low EVOO period among participants who started with the high EVOO (*p* = 0.01). In participants who started with the low EVOO, the positive affect change from baseline was greater during this first period compared to their second four weeks during high EVOO (*p* = 0.03), as well as compared to the high-to-low group over the same time period (*p* = 0.006). A greater decrease in perceived stress was reported in the low-to-high group over their second four weeks compared to the first four weeks (*p* = 0.03).

As shown in Table 2, two of the eight SF-36 scales (energy and general health) increased, while the remaining, except for physical functioning, changed in suggested positive directions but did not reach statistical significance (Table 2, Supplemental Figure S1).

## 4. Discussion

This investigation compared the effects of participating in a vegan group culinary medicine teaching kitchen intervention on psychological health among adults at risk for heart disease. Perceived stress and negative affect decreased, while positive affect progressively increased with improvements in energy/fatigue and general health-related quality-of-life measures. These results provide evidence of the potential for culinary medicine interventions as strategies for enhancing health and well-being.

**Figure 2:**
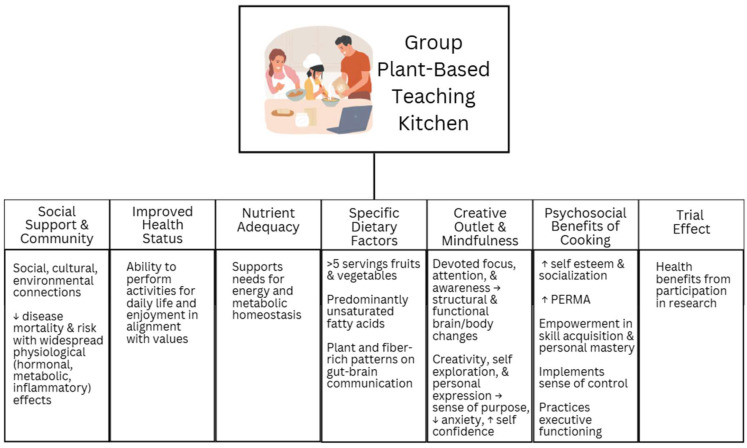
Potential mechanisms and contributing factors for improvements in psychological outcomes through participation in the Recipe for Heart Health culinary medicine intervention: Social Support and Community [28,49]; Improved Health Status [39]; Nutrient Adequacy [40]; Specific Dietary Factors [11,16,50,51,52,53]; Creative Outlet and Mindfulness [38]; Psychosocial Benefits of Cooking [31]; Trial Effect [54]. Up arrow correspond to increased; down arrows indicate decreased.

Many interconnected factors may have contributed towards mood and quality of life changes, including social support, overall improved health status, nutrient adequacy, specific dietary components within plant-rich patterns, facilitation of creativity, mindfulness, meditation, proposed psychosocial benefits connected to culinary interventions, and participation in a clinical trial (Figure 2). As meaningful relationships are recognized to be among the strongest predictors of disease and a public health priority [47], culinary interventions may provide an avenue for cultivating community with support from peers and healthcare providers, health coaches, or nutrition educators. Combined with inadequate nutrient intake, psychological and physical stress may additionally lead to micronutrient depletion and nutritional deficiencies, which can further impair health status [48].

In this study, mood-related changes coincided with improvements in metabolic factors [39] and nutrition intake, which enhanced diet quality affected by the intake of whole plant foods [40]. Current evidence suggests that healthful plant-based eating patterns may enhance measures of happiness while aiding in the prevention and management of mental health disorders, including depression and anxiety [12,13,23,24]. Hypothesized mechanistic factors include a range of interconnected hormonal, inflammatory, and neural pathways, encompassing communication between nutritional intake and microbiota (gut–brain axis), though further research is needed to elucidate mechanisms [50,55]. Combined with benefits of increased fruit and vegetable intake, the reduction of processed meat intake and potentially inflammatory animal-based compounds may additionally support psychological health [25,56]. Our study results contribute towards elucidating conflicting evidence on effects of the extent of exclusion of animal foods [22,57], which may vary considering other lifestyle stressors, predisposing factors, or the presence of disordered eating correlated to an increased risk of anxiety and depression. Extra virgin olive oil, also proposed as a component in Mediterranean diets to affect mood, appeared to somewhat contrast prior beneficial evidence, though possibly related to the volume advised to consume (4 tablespoons daily); this amount is consistent with guidance in Mediterranean patterns, though is higher than typically consumed in US populations [58].

Few studies have examined the psychosocial impacts of cooking interventions with similar results. Beneficial impacts on well-being and psychological health have been reported in a 64 h culinary medicine, physical activity, nutrition, and stress relief intervention among patients with cancer (20% reduction in stress and 54% increase in quality of life measures) [59], a randomized trial consisting of 12 weekly 30 min one-on-one telemedicine culinary coaching sessions in adults with overweight and obesity [60], and pilot CM studies [35,61,62]. Another randomized controlled trial of Coping with Cancer in the Kitchen, an 8-week CM program based on American Institute for Cancer Research recommendations, also suggested improved quality of life, lower psychological distress, and reduced fatigue [60]. To date, we are unaware of any other program or published evaluation of a virtual, multimodal group plant-based culinary medicine intervention with a focus on CVD prevention and psychosocial health.

Beyond enhanced nutritional status, additional features of cooking interventions may positively influence psychosocial health. In a systematic review evaluating cooking interventions, Farmer et al. reported potential benefits for confidence and self-esteem, socialization, quality of life, and mood and affect even without dietary changes [30]. Influence on these psychosocial outcomes may be explained by improvements in executive functioning through culinary related tasks, pleasurable memories, skill acquisition that boosts self-efficacy, enhanced nutrition status, and social relationship development [30,31,38].

The interpretation of the findings of this study should consider strengths and weaknesses. As mood and quality of life outcomes in this paper were exploratory, these variables were not the basis for power calculations, and values were not adjusted for multiple comparisons. Thus, significance should be confirmed in future investigations. While previous culinary medicine and teaching kitchen studies have suggested a beneficial role in psychological well-being, this study did not have a control group to compare results in non-participating individuals. With a primarily cohesive study sample of white women interested in cooking, additional trials of multiple populations are needed. Study strengths included the novel evaluation of virtual culinary medicine programming in a clinical population with widely used validated survey instruments, low attrition with high adherence and attendance (all participants with complete data attended all sessions), and comprehensive intervention support. While teaching kitchens are progressively growing, variation in data collection, inclusion of extensive virtual culinary resources/support in group and individual formats, and defined evaluation of outcomes (clinical and behavioral) has been limited in previous investigations. The Recipe for Heart Health study developed and demonstrated a unique application of culinary/lifestyle medicine in a clinical population that maintained high participant engagement for the full study duration.

Possible future applications of this model may be scalable through avenues within Food is Medicine and teaching kitchen initiatives [63,64]. While efforts are currently devoted towards establishing options for insurance coverage for these services as part of clinical care or public health [37,65], these vary by location in costs and delivery format. Offering a virtual or hybrid format, ideally that maintains connections to clinical or academic institutions, may allow for enhanced accessibility that integrates online on-your-own time and live sessions with support provided by trained or specialized professionals across multiple disciplines (e.g., dietitians, chefs, physicians, nurses, physical therapists, psychologists, personal trainers, occupational therapists, and researchers). Moreover, culinary medicine may be suitable for enhancing professional clinical care and telehealth services in a variety of formats.

## 5. Conclusions

This study evaluated the psychological outcomes of a multi-modal lifestyle behavior focused virtual culinary medicine teaching kitchen intervention in clinical patients at risk for heart disease. Participation led to suggested improvements in mood, stress, and quality of life. Multiple factors, including peer and clinician social support, improved health, nutrient adequacy, fiber-rich whole plant foods, psychosocial effects of cooking interventions, and research study participation, may have contributed towards results. The Recipe for Heart Health teaching kitchen model and advised dietary pattern may help inform future investigations. As a part of clinical care, culinary medicine may positively influence psychological health with potential beneficial implications on disease risk, well-being, and therapeutic treatment approaches. Early findings of suggested benefits warrant further research to evaluate effects on psychological and cognitive well-being.

## Figures and Tables

**Table 1:** Baseline characteristics of Recipe for Heart Health study population by randomization.

Characteristic	All (n = 40)	High to Low EVOO (n = 22)	Low to High EVOO (n = 18)
Age, years	64.4 ± 8.6	65.5 ± 6.3	63.0 ± 10.9
Sex, female, n (%)	30 (75%)	14 (64%)	16 (89%)
Primarily responsible for cooking meals, n (%)	32 (90%)	17 (77.3%)	15 (83.3%)
Primarily responsible for grocery shopping, n (%)	37 (92.5%)	20 (90.9%)	17 (94.4%)
Race/ethnicity, n (%)			
African American or Black	9 (22.5%)	5 (22.7%)	4 (22.2%)
Asian or Pacific Islander	1 (2.5%)	0 (0%)	1 (5.5%)
Hispanic/LatinX	1 (2.5%)	1 (4.5%)	0 (0%)
Non-Hispanic White	29 (72.5%)	15 (68.1%)	14 (77.7%)
Other	2 (5.0%)	2 (9.0%)	0 (0%)
Highest level of education achieved, n (%)			
High school degree	1 (2.5%)	0 (0%)	1 (5.6%)
Some college	13 (32.5%)	7 (31.8%)	6 (33.3%)
College degree	11 (27.5%)	6 (27.3%)	5 (27.8%)
Some post-graduate degree	2 (5.0%)	1 (4.5%)	1 (5.6%)
Post-graduate degree	13 (32.5%)	8 (36.4%)	5 (27.8%)
Income, n (%)			
USD 10,000–25,000	3 (7.5%)	0 (0%)	3 (16.7%)
USD 25,000–50,000	6 (15%)	5 (22.7%)	1 (5.6%)
USD 50,000–75,000	5 (12.5%)	2 (9.1%)	3 (16.7%)
USD 75,000–100,000	7 (17.5%)	3 (13.6%)	4 (22.2%)
USD 100,000–150,000	6 (15%)	5 (22.7%)	1 (5.6%)
USD > 150,000	10 (25%)	7 (31.8%)	3 (16.7%)
Prefer not to answer	3 (7.5%)	0 (0%)	3 (16.7%)
Children in household, n	1 (2.5%)	0 (0%)	1 (5.6%)
Reported daily sleep, hours			
About 5 h	4 (10%)	4 (18.2%)	0 (0%)
About 6 h	12 (30%)	5 (22.7%)	7 (38.9%)
About 7 h	15 (37.5%)	8 (36.4%)	7 (38.9%)
About 8 h	8 (20%)	4 (18.2%)	4 (22.2%)
More than 8 h	1 (2.5%)	1 (4.5%)	0 (0%)

**Table 2:** Short-form 36 health-related quality of life subscales at baseline and post Recipe for Heart Health intervention.

SF-36 Scale	Baseline	Post Intervention	Mean Difference	*p*-Value
Physical functioning	79.6 ± 3.4	78.8 ± 3.7	−0.9 ± 2.1	0.943
Role limitations due to physical health	81.3 ± 5.1	76.9 ± 5.6	−4.4 ± 4.4	0.311
Role limitations due to emotional problems	85.0 ± 5.2	90.0 ± 3.8	+5.0 ± 3.3	0.170
Energy/fatigue	58.4 ± 3.5	65.1 ± 3.0	+6.8 ± 2.3	0.006
Emotional well-being	78.8 ± 2.9	75.9 ± 2.2	+3.0 ± 1.8	0.128
Social functioning	72.8 ± 2.6	75.9 ± 2.2	+3.1 ± 1.6	0.079
Pain	71.4 ± 3.3	69.6 ± 4.2	−1.8 ± 2.1	0.340
General health	69.1 ± 2.7	73.2 ± 2.2	+4.1 ± 1.9	0.020

Values represent mean ± SEM. *p* values obtained from paired *t*-tests. SF-36, 36-Item Short-Form Survey.

## Data Availability

Data are available from the authors upon reasonable request. The data are not publicly available due to privacy reasons.

## Supplementary Materials

The following supporting information can be downloaded at: https://www.mdpi.com/article/10.3390/nu17081357/s1. Figure S1. Mean change in quality-of-life survey scale scores from baseline to post-intervention.
