# Supplementary material for: Influence of a Virtual Plant-Based Culinary Medicine Intervention on Mood, Stress, and Quality of Life Among Patients at Risk for Cardiovascular Disease
Source: Nutrients. 2025 Apr 16;17(8):1357. doi: 10.3390/nu17081357 (PMC12030687; doi:10.3390/nu17081357)

SUPPLEMENTARY MATERIALS

Figure S1. Mean change in quality-of-life survey scale scores from baseline to post-intervention. Data are mean+SEM. Paired t-tests compared pre-post survey scores for each SF-35 subscale. *P<0.05, **P<0.01. SF-36, 36-Item Short-Form Survey.

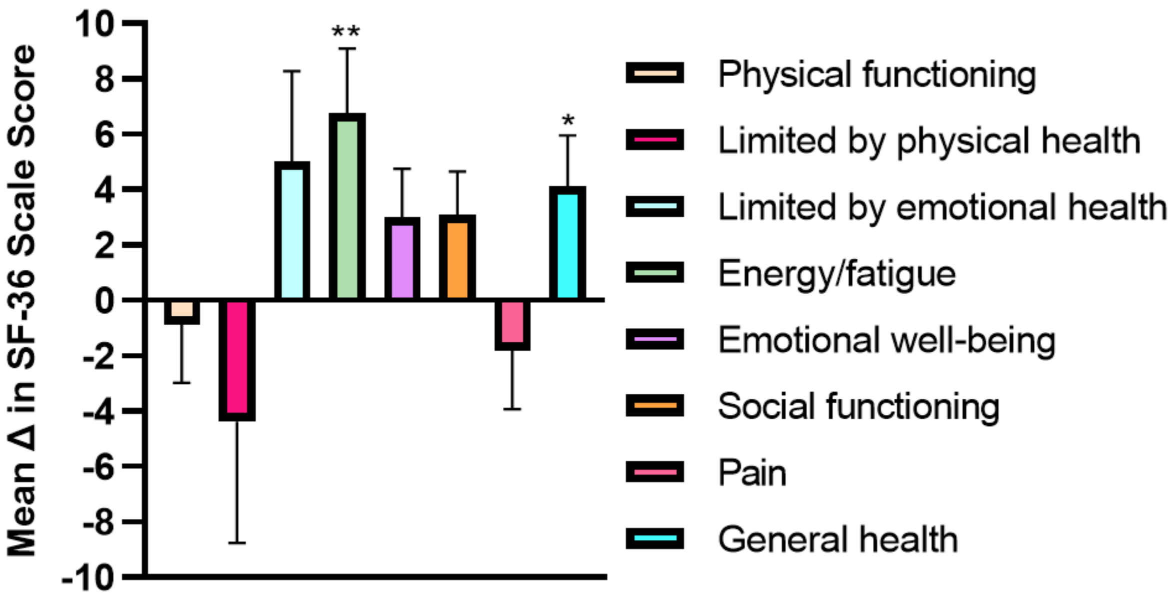

Supplement: Supplementary file 1 [file nutrients-17-01357-s001.zip › nutrients-3523090-supplementary.pdf]
